# Genetic variation of low-to-medium-affinity Fc-gamma receptors in Guillain-Barré syndrome

**DOI:** 10.1007/s00415-025-13216-8

**Published:** 2025-07-01

**Authors:** Sander J. van Tilburg, Selin Koçer, Judy Geissler, Wouter van Rijs, Anne P. Tio-Gillen, Michael W. T. Tanck, Willem-Jan R. Fokkink, Pieter A. van Doorn, Bart C. Jacobs, Sietse Q. Nagelkerke, Ruth Huizinga

**Affiliations:** 1 Department of Immunology, Erasmus MC, University Medical Center Rotterdam, Dr. Molewaterplein 40, 3015 GD Rotterdam, The Netherlands; 2 Department of Blood Cell Research, Sanquin Research Institute, University of Amsterdam, Amsterdam, The Netherlands; 3 Department of Neurology, Erasmus MC, University Medical Center Rotterdam, Rotterdam, The Netherlands; 4 Department of Epidemiology and Data Science, University of Amsterdam, Amsterdam UMC, Amsterdam, The Netherlands; 5 Department of Pediatric Immunology, Rheumatology and Infectious Diseases, Emma Children’s Hospital, Amsterdam UMC, University of Amsterdam, Amsterdam, The Netherlands

**Keywords:** Fc-receptors, Immune-mediated neuropathy, Polymorphisms, Autoantibodies, CNV

## Abstract

**Introduction:**

Fc-gamma receptors (FcγRs) are important for the effector functions of immunoglobulin G (IgG) and are therefore expected to play a role in the pathophysiology of Guillain-Barré syndrome (GBS). The *FCGR2/3* locus, which encodes low-to-medium-affinity FcγRs, contains extensive genetic variation. We hypothesized that genetic variation in the *FCGR2/3* locus influences GBS susceptibility, muscle weakness, outcomes, and the pharmacokinetics of intravenous immunoglobulin (IVIg).

**Methods:**

Copy number variation and single nucleotide polymorphisms in the *FCGR2/3* locus were studied using multiplex ligation-dependent probe amplification (MLPA). The study cohort consisted of 467 GBS patients and 919 healthy controls of European descent. Severe weakness was defined as an MRC sum score < 40 at nadir. The increase in serum IgG one or two weeks after start of IVIg treatment was determined.

**Results:**

No significant associations were found between genetic variation in the *FCGR2/3* locus and susceptibility to GBS. However, in patients with an antecedent *Campylobacter jejuni* infection, a higher frequency of three or more *FCGR3A* copies was observed compared to healthy controls (p = 0.023). *FCGR3A* copy numbers were also associated with more severe disease (OR = 2.02; 95% CI = 1.00–4.12), even after correcting for age and positive *C. jejuni* serology. No association was found between *FCGR2/3* variants and the ability to walk unaided in time-to-event analyses. In addition, the pharmacokinetics of IVIg were not affected by genetic variation in the *FCGR2/3* locus.

**Conclusion:**

Overall, *FCGR2/3* polymorphisms are not associated with susceptibility to GBS or response to IVIg treatment. However, associations may exist in specific subgroups, as demonstrated in patients with a preceding *C. jejuni* infection who more frequently carry a duplication in *FCGR3A*.

**Supplementary Information:**

The online version contains supplementary material available at 10.1007/s00415-025-13216-8.

## Introduction

Guillain-Barré syndrome (GBS) is an acute immune-mediated polyradiculoneuropathy. Most patients report symptoms of a preceding infection before the development of rapidly progressive weakness. Case–control and observational studies have identified several infectious triggers associated with GBS, including *Campylobacter jejuni, Mycoplasma pneumoniae,* cytomegalovirus*,* Zika virus*,* and hepatitis E virus [1–4]. It is presumed that antibodies induced to combat these microbes target epitopes that closely resemble gangliosides in the nerve. This concept of molecular mimicry is best exemplified in *C. jejuni*-associated GBS, where antibodies raised against *C. jejuni* lipo-oligosaccharide cross-react with GM1a on neuronal and Schwann cell membranes, resulting in complement deposition [5]. In about 60% of all patients, anti-ganglioside antibodies can be detected in the serum [6, 7]. However, only a small fraction of infected individuals develop GBS [8, 9], suggesting that other factors, genetic or environmental, play a role in the susceptibility to the disease.

Intravenous immunoglobulin (IVIg) is a proven, effective treatment for GBS [10]. However, the response to IVIg varies significantly between patients. Patients with rapid clearance of IVIg have worse clinical outcomes, suggesting that these individuals may benefit from alternative dosing regimens [11]. The exact mechanism underlying the effectiveness of IVIg in patients with GBS remains unknown, but both F(ab’)_2_-dependent and Fc-dependent modes of action are implicated [12]. The major receptors for the Fc domain of immunoglobulin G (IgG) are the Fc-gamma receptors (FcγRs), which play a crucial role in regulating immune responses. In GBS, anti-ganglioside antibodies are known to interact with FcγR in vitro [13]. In addition, activating FcγRs promote nerve inflammation in a passive transfer mouse model [14]. IVIg is thought to confer protection by blocking the binding of anti-ganglioside antibodies to these activating FcγRs, by modulating the inhibitory FcγRIIb on leukocytes, and by increasing the clearance of pathological antibodies by blocking the neonatal Fc Receptor (FcRn) [15, 16]. Given the role of FcγRs in the pathophysiology of GBS and the efficacy of IVIg, these receptors are of particular interest in understanding the susceptibility to disease and the response to IVIg.

FcγRs can be subdivided into high-affinity (FcγRI) and low-to-medium-affinity FcγRs (FcγRII and FcγRIII). The *FCGR2/3* locus, which encodes for the FcγRII and FcγRIII receptors, contains extensive genetic variation, including single nucleotide polymorphisms (SNPs) and copy number variation (CNV) [17]. Many of these genetic variations have demonstrated functional relevance and have been associated with auto-immune diseases and inflammatory neuropathies [18–22]. However, until now, studies in GBS have not accounted for the full complexity of this locus.

The aim of this study is to assess whether genetic variation in the *FCGR2/3* locus is associated with susceptibility, muscle weakness, outcomes, and the kinetics of IVIg in patients with GBS. We utilized multiplex ligation-dependent probe amplification (MLPA) to extensively genotype the *FCGR2/3* locus, enabling the determination of relevant SNPs as well as CNVs.

## Materials and methods

### Study participants

Patients of European descent fulfilling the diagnostic criteria for GBS [23] were included from previous clinical trials and studies conducted in the Netherlands [24–29]. Only patients with available DNA samples were eligible. Ethnicity was self-reported by patients, and ancestry and sex verification were performed by genotyping using the Infinium Global Screening Array MD version 3. Individuals of non-European descent were excluded. Approval was obtained from the Institutional Review Board of the Erasmus MC (MEC-2023-0100), and all patients provided written informed consent.

The control cohort consisted of a total of 919 healthy individuals from the Netherlands (n = 199), Austria (n = 478), Australia (n = 156), and the United Kingdom (n = 86), as previously published [20, 30]. All controls were of self-reported European descent.

### Clinical data

Clinical data were acquired from trial and study databases [24–29]. The GBS disability score (GBS-DS) was used to assess the functional status of patients, ranging from 0 (no disability) to 6 (death). A GBS-DS ≤ 2 corresponds to the ability to walk 10 m or more unaided. The Medical Research Council sum score (MRC-SS) was used to evaluate muscle strength in the arms and legs and ranges from 0 to 60. The MRC-SS was used to define disease severity, with mild disease classified as an MRC-sum score ≥ 40 at nadir and severe disease as an MRC-sum score < 40 at nadir. Electrophysiological studies, when conducted, were classified according to the Hadden criteria [31].

### Serology

The presence of IgG against GM1, GM2, GD1a, GD1b, and GQ1b was determined as described previously [32]. Serum antibodies indicative of a recent *C. jejuni* infection were assessed as described previously [33]. Total serum IgG concentrations were measured using turbidimetry. The ΔIgG concentrations at one and two weeks were calculated by subtracting the baseline serum IgG levels from those measured one and two weeks after IVIg administration (2 g/kg usually over 5 days).

### Multiplex ligation-dependent probe amplification (MLPA)

**Fig. 1:**
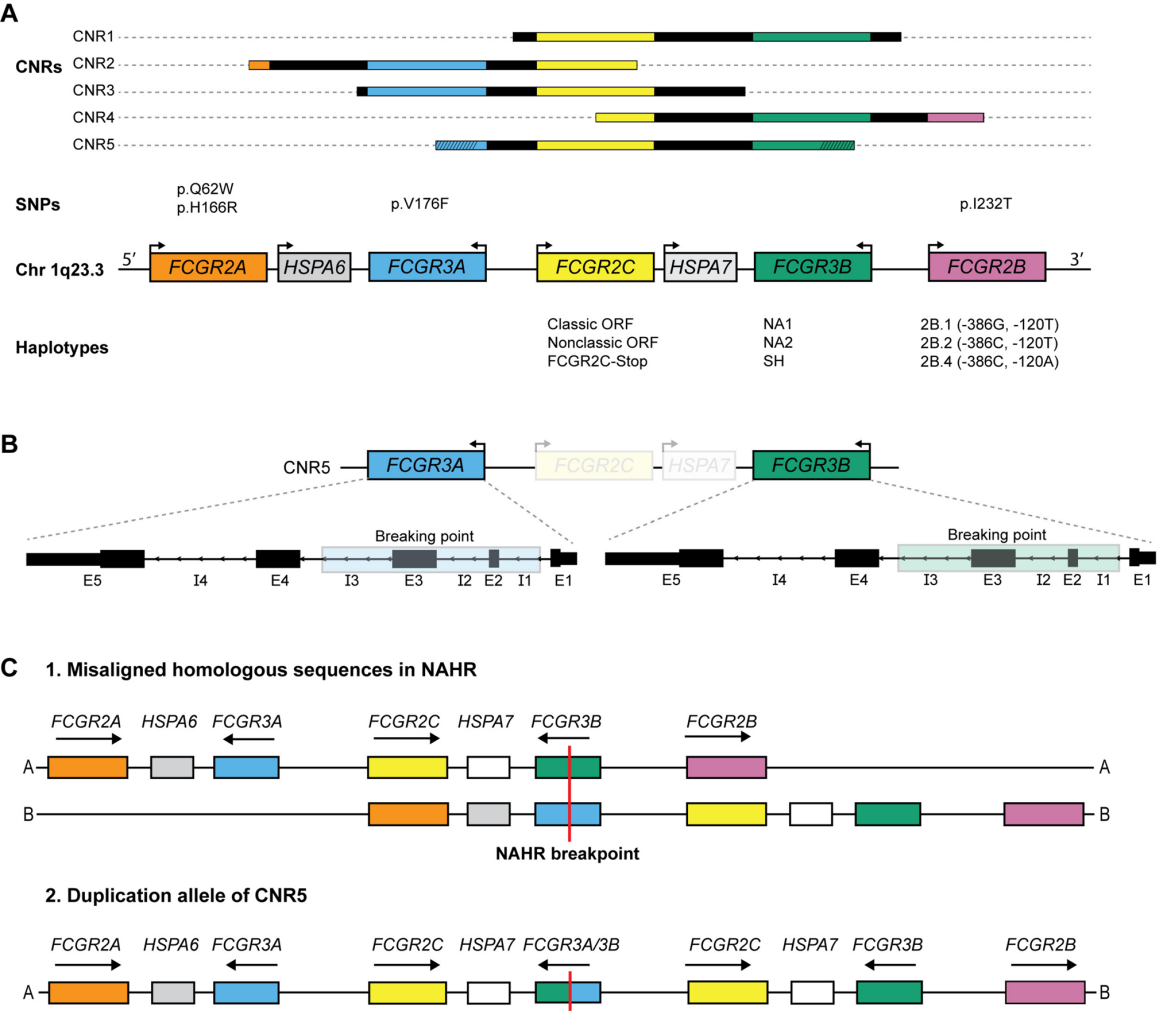
Genomic organization of the *FCGR2/3* locus at 1q23.3. **A** Copy number variation regions (CNRs) are identified at specific breakpoints, with each region encompassing a series of genes marked by matching colors. CNR5 denotes a novel CNR [51]. CNR1 and CNR4 are not separated due to the limitations of our method to distinguish between them. Gene orientations are indicated by arrows. Genes are not drawn to scale. All functional SNPs and haplotypes identified in this study are displayed for their respective genes. NA1, NA2, and SH are haplotypes determined by six SNPs. **B** Exact location of the breaking points in CNR5 are not known, but are within the highlighted area. **C** Schematic overview of the theoretical duplication allele of CNR5 leading to a chimeric *FCGR3A/3B* gene. Abbreviations: *CNR* copy number variation region, *SNP* single nucleotide polymorphism, *Chr* chromosome, *ORF* open-reading frame, *NAHR* nonallelic homologous recombination

Genomic DNA was extracted from peripheral blood following standard procedures. The MLPA assay (MRC Holland, Amsterdam, The Netherlands) was performed according to the manufacturer’s instructions and has been described in detail previously [18, 20]. In brief, probes were included to detect the following SNPs and haplotypes in the *FCGR2/3* locus: *FCGR2A* (p.Q62W, p.H166R)*, FCGR2B* (p.I232T), *FCGR2C* haplotypes (classic/non-classic/stop)*, FCGR3A* (p.V176F)*,* and *FCGR3B* haplotypes (NA1/NA2/SH) (Fig. 1)*.* MLPA probes were specifically designed to identify CNV regions (Fig. 1). *FCGR2C* consists of three haplotypes; classic *FCGR2C*-ORF with an open reading frame (ORF) (p.57Q), *FCGR2C*-Stop (p.57Stop), and the nonclassic *FCGR2C*-ORF with a splice mutation at the exon 7-intron 7 border (c.798 + 1A > G), resulting in an almost complete lack of expression. Promoter regions of *FCGR2B* were defined as 2B.1 ( –386G, –120T), 2B.2 ( –386C, –120T) and 2B.4 ( –386C, – 120A), these promoter haplotypes were allocated as described previously [20]. In each experiment, data were assessed in relation to three genotyped controls representing all relevant genetic variations as a reference.

### Statistics

SNPs and CNVs in the *FCGR2/3* locus were compared between patients with GBS and healthy controls using Fisher’s exact test, applying a correction for multiple testing (Bonferroni) when non-corrected p-values were significant. Logistic regression analysis was used to assess the association between SNPs and copy numbers of CNRs in the *FCGR2/3* locus and the severity of weakness. For analyses with corrections for age and *C. jejuni* serology, a cohort without missing values was used (n = 453). Survival analysis (log-rank test for trend) was performed to analyze the relationship between SNPs and CNVs in the *FCGR2/3* locus and clinical outcomes (time to walk unaided). Associations between genotypes in the *FCGR2/3* locus and ΔIgG levels one week and two weeks after start of IVIg treatment were assessed by Kruskal–Wallis rank sum test. P-values less than 0.05 were considered statistically significant. Analyses were performed in R (version 4.2.1).

## Results

### Patient cohort

A total of 477 patients with GBS were genotyped. After verifying sex and ancestry, 10 patients of non-European descent were excluded. The final cohort consisted of 467 patients. Patient characteristics of the study cohort are described in Supplemental Table 1.

**Table 1:** Frequency of SNPs and CNV at the FCGR2/3 locus in cases and controls

Variant	GBS cases (%)	CJ+ GBS (%)	AGA+ GBS (%)	Controls (%)	*P* GBS vs controls	*P* CJ+ vs controls	*P* AGA+ vs controls
*FCGR2A* p.Gln62Trp					0.7	0.45	0.74
QQ	353 (75.6)	92 (73.0)	83 (78.3)	713 (77.6)			
QW	108 (23.1)	32 (25.4)	23 (21.7)	194 (21.1)			
WW	6 (1.3)	2 (1.6)	0 (0)	12 (1.3)			
*FCGR2A* p.His166Arg					0.54	0.74	0.20
HH	126 (27.0)	33 (26.2)	25 (23.6)	269 (29.3)			
HR	236 (50.5)	65 (51.6)	52 (49.1)	463 (50.4)			
RR	105 (22.5)	28 (22.2)	29 (27.4)	187 (20.3)			
*FCGR2B promoter*					0.09	1	0.37
0 2B.4	372 (79.7)	104 (82.5)	92 (86.8)	748 (81.4)			
1 2B.4	93 (19.9)	21 (16.7)	14 (13.2)	157 (17.1)			
2 2B.4	2 (0.4)	1 (0.8)	0 (0)	14 (1.5)			
*FCGR2B* p.Ile232Thr					0.026 (0.34)	0.27	0.19
II	382 (81.8)	104 (82.5)	89 (84.0)	697 (75.8)			
IT	80 (17.1)	20 (15.9)	16 (15.1)	201 (21.9)			
TT	5 (1.1)	2 (1.6)	1 (0.9)	21 (2.3)			
*FCGR2C* NC-ORF					0.14	0.96	0.82
0 NC ORF	446 (95.5)	117 (92.9)	101 (95.3)	853 (92.8)			
1 NC ORF	9 (1.9)	5 (4.0)	3 (2.8)	33 (3.6)			
2 NC ORF	12 (2.6)	4 (3.2)	2 (1.9)	33 (3.6)			
*FCGR2C* ORF					0.42	0.32	0.52
0 ORF	351 (75.2)	91 (72.2)	88 (83.0)	721 (78.5)			
1 ORF	110 (23.6)	33 (26.2)	18 (17.0)	184 (20.0)			
2 ORF	6 (1.3)	2 (1.6)	0 (0)	13 (1.4)			
3 ORF	0 (0)	0 (0)	0 (0)	1 (0.1)			
*FCGR3A* p.Val176Phe					0.26	0.14	0.15
0 V (F, FF, FFF, FFFF)	185 (39.6)	43 (34.1)	46 (43.4)	386 (42.0)			
1 V (V, VF, VFF)	223 (47.8)	59 (46.8)	39 (36.8)	403 (43.9)			
2 V (VV, VVF, VVFF)	56 (12.0)	23 (18.3)	20 (18.9)	128 (13.9)			
3 V (VVV)	3 (0.6)	1 (0.8)	1 (0.9)	2 (0.2)			
*FCGR3B* NA1/NA2					0.87	0.34	0.48
0 NA1	188 (40.6)	56 (44.4)	50 (48.1)	373 (40.6)			
1 NA1	213 (46.0)	49 (38.9)	42 (40.4)	430 (46.8)			
2 NA1	62 (13.4)	20 (15.9)	12 (11.5)	114 (12.4)			
3 NA1	0 (0)	0 (0)	0 (0)	2 (0.2)			
*FCGR3B* SH					0.15	0.5	0.47
0 SH	450 (96.4)	122 (96.8)	103 (97.1)	874 (95.1)			
1 SH	16 (3.4)	4 (3.2)	3 (2.8)	45 (4.9)			
2 SH	1 (0.2)	0 (0)	0 (0)	0 (0)			
CNR1 (*FCGR2C* + *FCGR3B*)					0.26	0.075	0.10
0 copies	2 (0.4)	1 (0.8)	1 (0.9)	1 (0.1)			
1 copy	41 (8.8)	14 (11.1)	12 (11.3)	60 (6.5)			
2 copies	375 (80.3)	96 (76.2)	85 (80.2)	768 (83.6)			
3 copies	43 (9.2)	15 (12.0)	7 (6.6)	83 (9.0)			
4 copies	6 (1.3)	0 (0)	1 (0.9)	7 (0.8)			
CNR2 (*FCGR2C* + *FCGR3A*)					0.17	0.009 (0.12)	0.064
1 copy	3 (0.6)	0 (0)	1 (0.9)	11 (1.2)			
2 copies	432 (92.5)	113 (89.7)	95 (89.6)	866 (94.2)			
3 copies	30 (6.4)	11 (8.7)	9 (8.5)	41 (4.5)			
4 copies	2 (0.4)	2 (1.6)	1 (0.9)	1 (0.1)			
CNR3 (*FCGR2C* + *FCGR3A*)					0.19	0.014 (0.18)	0.055
2 copies	463 (99.1)	123 (97.6)	104 (98.1)	917 (99.8)			
3 copies	4 (0.9)	3 (2.4)	2 (1.9)	2 (0.2)			
*FCGR3A*					0.083	0.0018 (0.023)	0.030 (0.39)
1 copy	3 (0.6)	0 (0)	1 (0.9)	11 (1.2)			
2 copies	428 (91.6)	110 (87.3)	93 (87.7)	864 (94.0)			
3 copies	34 (7.3)	14 (11.1)	11 (10.4)	43 (4.7)			
4 copies	2 (0.4)	2 (1.6)	1 (0.9)	1 (0.1)			

GBS patients are stratified for antecedent infection with Campylobacter jejuni (CJ+) or presence of anti-ganglioside IgG (AGA+). P-values in brackets indicate the corrected P-value after Bonferroni correction (only shown when p-value before correction is significant). *CI* Confidence interval, *CNR* copy number region, *ORF* open reading frame

### SNPs and CNVs in the FCGR2/3 locus do not generally increase susceptibility to GBS

The genotype frequencies of known functional SNPs and CNVs in the *FCGR2/*3 locus (Fig. 1) are shown in Table 1 for patients with GBS and controls. The CNV of *FCGR3A* was analyzed separately because CNR2 and CNR3 both include *FCGR3A*. In four patients *FCGR3B*-NA1/NA2 could not be assessed. One patient, with a preceding cytomegalovirus infection, was identified with three copies of parts of both *FCGR3A* and *FCGR3B* and three copies of the entire *FCGR2C* and *HSPA7* genes, suggestive of a novel CNR (CNR5, Fig. 1B–C). All genotypes were in Hardy–Weinberg equilibrium. The *FCGR2B*-p.I232T genotype frequency differed between patients and controls (Fisher’s exact, p = 0.026) with a higher frequency of patients with homozygous *FCGR2B*-p.232I (81.8% in patients vs. 75.8% in controls). However, no significant difference was found after correction for multiple testing (Fisher’s exact, p_cor_ = 0.21; Table 1). The frequency of other SNPs and CNVs were not different between patients with GBS and healthy controls.

### FCGR3A copy numbers are associated with *C. jejuni* positive GBS

Patients were then stratified based on evidence of a recent infection with *C. jejuni*, which is the most common antecedent infection in GBS. No significant differences were found in SNPs between *C. jejuni* positive patients with GBS and controls (Table 1) or *C. jejuni* negative patients and controls (data not shown). For CNVs, duplications of CNR2 (*FCGR3A* + *FCGR2C*) and CNR3 (*FCGR3A* + *FCGR2C*) were more prevalent in *C. jejuni* seropositive cases than in controls (p = 0.009 and p = 0.014 respectively; p_cor_ = 0.12 and p_cor_ = 0.18, respectively). CNV of *FCGR3A* significantly differed between *C. jejuni* positive GBS patients and controls, with a higher prevalence of a duplication of *FCGR3A* in *C. jejuni* positive cases (p = 0.0018, p_cor_ = 0.023; Table 1). Similarly, a significant difference was observed between *C. jejuni* positive and negative cases (p = 0.022), but not after correction (p_cor_ = 0.29).

Next, we compared the frequency of SNPs and CNVs in the *FCGR2/3* locus between controls and patients positive for anti-ganglioside IgG antibodies (Table 1). This cohort consisted of 106 patients with anti-GM1, anti-GM2, anti-GD1a, anti-GD1b and/or anti-GQ1b IgG antibodies (reactivities specified in Supplemental Table 1). In line with our previous findings, a duplication in *FCGR3A* was significantly more frequent in anti-ganglioside IgG positive cases versus controls (p = 0.030), but this did not remain significant after correction for multiple testing (p_cor_ = 0.39).

No differences were found in frequencies of SNPs and CNVs in the *FCGR2/3* locus between patients with the axonal or demyelinating subtype of GBS and controls (data not shown).

### Clinical association between muscle weakness and genetic variation in FCGR2/3 locus

Within the complete GBS cohort associations of SNPs and CNVs in the *FCGR2/3* locus with severe GBS (defined as MRC-ss at nadir ≤40) were determined (Table 2). A duplication (3 copies) of *FCGR3A* was found to be associated with severe weakness (OR = 2.02, CI = 1.00–4.12, p = 0.049). After correction for age and preceding *C. jejuni* infection, the association of *FCGR3A* with disease severity remained (OR = 2.07, CI = 1.02–4.25, p = 0.043). In addition, a trend for an association of CNR2 duplication with severe GBS was observed (OR = 2.04, CI = 0.97 – 4.35, p = 0.054). Other SNPs and CNVs were not significantly associated with severe GBS.

**Table 2:** Frequency of SNPs and CNVs at the FCGR2/3 locus in GBS patients with severe disease compared to mild disease

Variant	Severe disease n = 173 (%)	Mild disease n = 284 (%)	Odds ratio (95% CI)
CNR1 (*FCGR2C* + *FCGR3B*)			
0 copies	2 (1.2)	0	NA
1 copy	18 (10.4)	25 (8.5)	1.41 (0.73–2.69)
2 copies	134 (77.5)	241 (82.0)	Reference
3 copies	17 (9.8)	27 (9.2)	1.18 (0.61–2.23)
4 copies	2 (1.2)	4 (1.4)	0.90 (0.12–4.67)
CNR2 (*FCGR2C* + *FCGR3A*)			
1 copy	2 (1.2)	1 (0.3)	3.57 (0.34–77.3)
2 copies	155 (89.6)	277 (94.2)	Reference
3 copies	16 (9.2)	14 (4.8)	2.04 (0.97–4.35)
4 copies	0 (0)	2 (0.7)	NA
CNR3 (*FCGR2C* + *FCGR3A*)			
2 copies	171 (98.8)	292 (99.3)	Reference
3 copies	2 (1.2)	2 (0.7)	1.71 (0.20–14.34)
*FCGR3A*			
1 copy	2 (1.2)	1 (0.3)	3.59 (0.34–77.72)
2 copies	153 (88.4)	275 (93.5)	Reference
3 copies	18 (10.4)	16 (5.4)	2.02 (1.00–4.12)
4 copies	0	2 (0.7)	NA
*FCGR2A* p.Gln62Trp			
QQ	139 (80.3)	214 (72.8)	Reference
QW	32 (18.5)	76 (25.9)	0.65 (0.40–1.02)
WW	2 (1.2)	4 (1.4)	0.77 (0.11–4.00)
*FCGR2A* p.His166Arg			
HH	42 (24.3)	84 (28.6)	Reference
HR	92 (53.2)	144 (49.0)	1.28 (0.81–2.02)
RR	39 (22.5)	66 (22.4)	1.18 (0.69–2.03)
*FCGR3A* p.Val176Phe			
0 V (F, FF, FFF, FFFF)	71 (41.0)	114 (38.8)	Reference
1 V (V, VF, VFF)	82 (47.4)	141 (48.0)	0.93 (0.62–1.40)
2 V (VV, VVF, VVFF)	18 (10.4)	38 (12.9)	0.76 (0.40–1.42)
3 V (VVV)	2 (1.2)	1 (0.3)	3.21 (0.30–69.85)
Classic *FCGR2C* ORF			
0 Classic ORF	137 (79.2)	214 (72.8)	Reference
1 Classic ORF	35 (20.2)	75 (25.5)	0.73 (0.46–1.14)
2 Classic ORF	1 (0.6)	5 (1.7)	0.31 (0.02–1.96)
Non-classic *FCGR2C* ORF			
0 Non-classic ORF	162 (93.6)	284 (96.6)	Reference
1 Non-classic ORF	4 (2.3)	5 (1.7)	1.40 (0.34–5.37)
2 Non-classic ORF	7 (4.0)	5 (1.7)	2.45 (0.77–8.41)
*FCGR3B* NA1/NA2			
0 NA1	77 (45.3)	111 (37.9)	Reference
1 NA1	72 (42.4)	141 (48.1)	0.74 (0.49–1.10)
2 NA1	21 (12.4)	41 (14.0)	0.74 (0.40–1.34)
*FCGR3B* SH			
0 SH	164 (94.8)	286 (97.3)	Reference
1 SH	9 (5.2)	7 (2.4)	2.24 (0.82–6.38)
2 SH	0 (0)	1 (0.3)	NA
*FCGR2B promoter*			
0 2B.4	139 (80.3)	233 (79.3)	Reference
1 2B.4	34 (19.7)	59 (20.1)	0.97 (0.60–1.54)
2 2B.4	0	2 (0.7)	NA
*FCGR2B* p.Ile232Thr			
II	134 (77.5)	248 (84.4)	Reference
IT	36 (20.8)	44 (15.0)	1.51 (0.93–2.46)
TT	3 (1.7)	2 (0.7)	2.78 (0.45–21.27)

CI including NAs are reported as NA. *CI* Confidence interval, *CNR* copy number region, *ORF* open reading frame

Next, we investigated whether genetic variation is associated with the time required for recovery. No significant association was observed between the time to regain the ability to walk unaided and genetic variation in the *FCGR2/3* locus.

### No association between ΔIgG and genetic variation

To evaluate the impact of genetic variation in the *FCGR2/3* locus on IgG clearance, the relationship between ΔIgG serum concentration and SNPs and CNVs was assessed. ΔIgG concentrations at one and two weeks after start of IVIg (2 g/kg) were calculated for 165 and 192 patients, respectively. Patients who received a second IVIg dose were excluded from the two-week analysis but included in the one-week analysis, as the IgG concentration was measured before the second dose was administered. No significant association was found between copy numbers or SNPs and IVIg kinetics when examining the one-week or two-week ΔIgG concentration (Supplemental Table 2).

## Discussion

This is the first study to capture the full complexity of the *FCGR2/3* locus in patients with GBS, including CNVs. The data demonstrate that; (1) there are no differences in frequencies of SNPs and CNVs between all patients with GBS and controls, (2) duplications in *FCGR3A* are more frequent in GBS patients with an antecedent *C. jejuni* infection compared to controls, (3) more copies of *FCGR3A* are associated with severe disease and (4) genetic variation at the *FCGR2/3* locus is not associated with ΔIgG serum concentrations.

No significant differences in the frequencies of SNPs and CNVs were found between unstratified patients with GBS and controls. Our data extend and align with previous studies, in which no associations were reported for SNPs in Bangladeshi patients or in a meta-analysis cohort consisting of UK, Dutch, and Norwegian patients with GBS [21, 34]. In contrast, other studies have observed associations with SNPs in *FCGR2A* and *FCGR3A* [35, 36]. Small group size, heterogeneity in patient subgroups, and differences in genetic backgrounds may explain these discrepancies. Variations in *FCGR2B* (encoding the inhibitory FcγRIIb), *FCGR2C* (encoding FcγRIIc, expressed in approx. 10% of individuals), as well as CNVs, have not been previously investigated in GBS. In contrast to other acute immune-mediated diseases such as Kawasaki disease and immune thrombocytopenia, we observed no association between these genetic variants and the development of GBS when correction for multiple testing was applied [20, 37]. Potentially, the contribution of effector mechanisms involved in humoral and cellular immunity may differ between and within these diseases. Together, these findings suggest that SNPs and CNVs in the *FCGR2/3* locus are not general genetic susceptibility factors for GBS but may be involved in susceptibility for GBS in specific subgroups of patients.

Our results suggest that CNV of *FCGR3A* influences susceptibility to develop GBS following an infection with *C. jejuni*, albeit in a relatively small subgroup of patients with a recent *C. jejuni* infection (n = 126). GBS is considered a post-infectious disease, with serological evidence of a recent infection identified in approximately 41–59% of patients, most commonly caused by *C. jejuni* in 30–32% of cases [1, 2]. Given that almost 100% of the general population has been exposed to *C. jejuni* infection by the age of 20 [38], but only 1 in 1000–2000 infected individuals develops GBS, other environmental and genetic factors must be involved. Previously, small studies have shown an association between genetic variation in HLA and TNF-α in GBS associated with *C. jejuni* [39–41]. Our study adds CNV in *FCGR3A* as a potential contributor to susceptibility for GBS after a *C. jejuni* infection. We did not find a statistically significant difference between *C. jejuni* positive and negative GBS patients after correction for multiple testing. A relatively small sample size may have limited the statistical power. In addition, the *C. jejuni* negative group is heterogenous and likely includes patients with other bacterial infections, such as *Mycoplasma pneumoniae* and *Haemophilus influenzae,* and viral infections or vaccinations. It is possible that genetic factors contributing to the development of GBS, such as *FCGR3A* CNV are shared between *C. jejuni* GBS and some of the other antecedent events subgroups.

Mechanistically, a gene-dosage effect of *FCGR3A* copy numbers on FcγRIIIa (CD16a) expression on natural killer (NK) cells was previously demonstrated, which correlated with the ability of NK cells to induce antibody-dependent cellular cytotoxicity (ADCC) [42]. As NK cells have been reported to be elevated in the cerebrospinal fluid of patients with GBS [43], we hypothesize that increased expression of FcγRIIIa on NK cells may lead to augmented anti-ganglioside antibody-mediated ADCC, resulting in peripheral nerve pathology. Besides NK cells, macrophages and subsets of monocytes also express FcγRIIIa, which are involved in antibody-dependent phagocytosis [44]. Of note, *FCGR3A* copy numbers were similarly increased in the subgroup of patients with anti-ganglioside IgG antibodies (11.7% of anti-ganglioside IgG positive patients had 3 or more copies vs. 12.7% of *C. jejuni* seropositive patients). One possible explanation for our finding is that increased expression of FcγRIIIa may promote anti-ganglioside antibody-mediated phagocytosis of myelin or axons. In line with this hypothesis, there is in vivo evidence that CNV in *FCGR3A* affects the efficiency of ADCC. A recent study showed that CNV in *FCGR3A* was associated with a higher likelihood of complete depletion of CD20 positive B-cells in the peripheral blood of patients treated with Rituximab [45]. The findings regarding *FCGR3A* copy numbers in relation to a recent *C. jejuni* infection should be validated in an independent cohort.

We found that patients with three copies of *FCGR3A* have increased odds of developing severe GBS. This association may be indirect since having > 2 copies of *FCGR3A* was also more prevalent in patients who developed GBS after a *C. jejuni* infection, and it is well-established that patients with a recent *C. jejuni* infection often experience more severe disease [1]. However, after correction for age and *C. jejuni* serology, *FCGR3A* remained significantly associated with increased muscle weakness. Previous studies in patients with GBS reported associations between severe disease and polymorphisms in *FCGR2A*, *FCGR3A*, and *FCGR3B* [21, 34, 36, 46]. However, we could not replicate any of these findings in our large cohort. Potentially, the discrepancies may be explained by the fact that CNVs were not accounted for in previous studies.

We did not observe a significant relationship between ΔIgG concentrations one or two weeks after a single course of IVIg administration and genetic variations in the *FCGR2/3* locus. However, it is important to note that we did not account for potential confounding factors, such as severe disease and concomitant methylprednisolone treatment, both of which have been shown to influence IgG clearance according to a recently developed pharmacokinetic model [47]. Although multiple SNPs are associated with increased binding affinity for various IgG subclasses [17, 48], most of these interactions require the prior formation of immune complexes, as FcγRII and -III are low-to-medium-affinity receptors that are unable to interact with IgG monomers [48]. In other diseases, such as chronic inflammatory demyelinating polyneuropathy, immune thrombocytopenia and Kawasaki disease, genetic variation was associated with favorable response to IVIg [22, 37, 49]. However, for Kawasaki disease, this finding could not be replicated in a larger cohort [50]. The absence of a relationship between genetic variation at the *FCGR2/3* locus and long-term clinical outcomes in our study may suggest that such genetic variation does not influence the response to IVIg in patients with GBS. Nevertheless, Fc-receptors in general may still influence the pharmacokinetics and pharmacodynamics of IVIg and other antibody-based therapies.

In the present study, we identified a unique case of a GBS patient with a preceding cytomegalovirus infection who had three copies of parts of *FCGR3A* and *FCGR3B*, along with three copies of the entire *FCGR2C* and *HSPA7* genes. Interestingly, a recent study described a deletion with breakpoints within *FCGR3A* and *FCGR3B*, resulting in a chimeric *FCGR3A/3B* gene [51]. This variant was found in an indigenous community in North-Eastern Quito, Ecuador. This new CNR with breakpoints within *FCGR3A* and *FCGR3B* was named ‘CNR5’. In contrast to the reported deletion, we observed a duplication with breakpoints within *FCGR3A* and *FCGR3B*, which, to our knowledge, has not been previously reported. This novel duplication is likely the result of nonallelic homologous recombination (NAHR) and, similar to the situation of duplications of CNR2 [52], could lead to an expressed chimeric FcγR. However, preliminary results using PCR showed no evidence for the presence of chimeric *FCGR3A/3B* transcripts in peripheral blood mononuclear cells, while normal *FCGR3A* was present (data not shown). The impact of the new CNR5 on FcγRIII function remains to be elucidated, but in view of the rarity of this variant, we do not expect it to play a large role in the pathophysiology of GBS.

This study has several limitations. First, while the control group consisted of individuals of European descent, the study cohort included only Dutch patients. Additionally, the relationship between genetic variation, muscle weakness, and outcomes could have been confounded by factors such as treatment, disease subtype, and other clinical or biological variables. Although the sample size is relatively large for a rare disease like GBS and compares favorably to previous studies, subgroup analyses remain limited. Interpretation of the results should therefore be approached with caution, as some subgroup sample sizes were small. Furthermore, associations with muscle weakness were borderline significant, and significance was lost after adjusting for other factors known to influence severity. This highlights the need for larger sample sizes or meta-analyses to validate these findings. Finally, as the cohort included only Caucasian individuals, validation studies in cohorts of other ethnicities are necessary.

In conclusion, our comprehensive genetic study using the MLPA technique provides no evidence for a role of the *FCGR2/3* locus in the general susceptibility of GBS. However, subgroup analyses suggest the involvement of *FCGR3A* duplication in susceptibility to GBS associated with a *C. jejuni* infection, indirectly leading to more severe disease. Future studies addressing the full complexity of the *FCGR2/3* locus in specific subgroups of patients with GBS will be key to better understanding the role of Fc-gamma receptors in the pathophysiology of GBS.

## Supplementary Information

Below is the link to the electronic supplementary material.

Supplementary file1 (DOCX 20 KB)

## Data Availability

In compliance with the General Data Protection Regulation, the source data cannot be made available to other researchers as patient approval has not been obtained for sharing coded data. Information about the analytic methods, syntax, and output files of statistical analyses will be made available by the corresponding author upon reasonable request.
