# Supplementary material for: Genetic variation of low-to-medium-affinity Fc-gamma receptors in Guillain-Barré syndrome
Source: J Neurol. 2025 Jul 1;272(7):487. doi: 10.1007/s00415-025-13216-8 (PMC12213970; doi:10.1007/s00415-025-13216-8)
Supplement: Supplementary file 1 — Supplementary file1 (DOCX 20 KB) [file 415_2025_13216_MOESM1_ESM.docx]

**Supplemental Table 1:** Baseline characteristics patient cohort

| **Characteristics** | **GBS cases  n = 467 (%)** |
| --- | --- |
| **Sex (n, %)** |  |
| Male | 279 (60%) |
| Female | 188 (40%) |
| **Age (years, IQR)** | 56 (40, 66) |
| **NCS (n, %)** |  |
| Normal | 6 (1.4%) |
| Demyelinating | 250 (60%) |
| Axonal | 27 (6.5%) |
| Equivocal | 123 (30%) |
| Inexcitable | 10 (2.4%) |
| NP or NA* | 51 |
| **Preceding infection (n, %)** |  |
| Diarrhea | 200 (43%) |
| URTI | 71 (15%) |
| Other | 43 (9.2%) |
| No URTI/Diarrhea | 81 (17%) |
| None | 72 (15%) |
| **Positive *C.jejuni* serology (n, %)** | 126 (28%) |
| Unknown | 15 |
| **MRC-SS at nadir (median, IQR)** | 44 (32 – 50) |
| **GBS-DS at nadir (median, IQR)** | 4 (3 – 4) |
| **Anti-ganglioside IgG (+/total n)** |  |
| GM1+ | 66/453 (14.6%) |
| GM2+ | 10/416 (2.4%) |
| GD1a+ | 23/453 (5.1%) |
| GD1b+ | 61/382 (16.0) |
| GQ1b+ | 12/416 (2.9) |

*NCS, nerve conduction studies; NP, not performed; URTI, upper respiratory tract infection; MRC-SS, Medical Research Council sum score; GBS-DS, GBS disability score; IQR, interquartile range (25^th^ – 75^th^ percentile)*

**Supplemental Table 2:** Association between CNV, SNPs and haplotypes in the *FCGR2/3* locus and ΔIgG concentration at one and two weeks. P-values are derived using the Kruskal-Wallis rank sum test.

| **Variant** | **ΔIgG one week**  **p-value** | **ΔIgG two weeks**  **p-value** |
| --- | --- | --- |
| **Copy number variation**  CNR1 (*FCGR2C* + *FCGR3B*) | 0.65 | 0.63 |
| CNR2 (*FCGR2C* + *FCGR3A*) | 0.44 | 0.40 |
| CNR3 (*FCGR2C* + *FCGR3A*) | 0.33 | 0.41 |
| *FCGR3A* | 0.40 | 0.39 |
| **SNP / Haplotypes** |  |  |
| *FCGR2A* p.Gln62Trp | 0.69 | 0.35 |
| *FCGR2A* p.His166Arg | 0.10 | 0.14 |
| *FCGR3A* p.Val176Phe | 0.89 | 0.08 |
| Classic *FCGR2C* ORF | 0.90 | 0.37 |
| Non-classic *FCGR2C* ORF | 0.70 | 0.057 |
| *FCGR3B* NA1/NA2 | 0.98 | 0.50 |
| *FCGR3B* SH | 0.80 | 0.15 |
| *FCGR2B* promoter | 0.70 | 0.85 |
| *FCGR2B* p.Ile232Thr | 0.23 | 0.24 |

*CNR, copy number region; FCGR, Fc gamma receptor; ORF, open reading frame; SNP, single nucleotide polymorphism*
